# High prevalence of periodontal disease and periodontopathogen colonization in adults with autism spectrum disorder: a pilot study

**DOI:** 10.3389/fmicb.2025.1552656

**Published:** 2025-02-28

**Authors:** Ludivine Berbé, Marie Machouart, Amandine Luc, Eliane Albuisson, Catherine Strazielle, Catherine Bisson

**Affiliations:** ^1^SIMPA, Université de Lorraine, Nancy, France; ^2^Département de parodontologie, CHRU-Nancy, Nancy, France; ^3^Département de parasitologie, CHRU-Nancy, Nancy, France; ^4^Département méthodologie, promotion et investigation, UMDS, CHRU-Nancy, Nancy, France; ^5^Service d’odontologie-Brabois adultes, CHRU-Nancy, Nancy, France

**Keywords:** autism spectrum disorder, periodontitis, periodontal parameters, periodontal bacteria, protozoa

## Abstract

**Introduction:**

Alteration of the oral microbiome could potentially play a role in the etiology of certain patients with Autism Spectrum Disorder (ASD), similar to the established link between gut microbiota dysbiosis and ASD. Most studies have assessed oral microbiota in children only and few have explored the oral flora composition in adults with ASD.

**Methods:**

In our study, periodontal and dental status was evaluated in 30 adults with ASD using appropriate indices. Oral microbiota samples were collected in crevicular fluid and supra-gingival plaque at inflamed sites in each patient and analyzed using PCR for bacteria and qPCR for protozoa. Demographic data, co-morbidities, medication and oral hygiene habits were also collected.

**Results:**

A total of 86.7% of the patients recruited suffering from severe ASD had periodontal disease and 67% had a high level of supra-gingival plaque. Two major periodontopathogens belonging to the red complex, *Treponema denticola* and *Tannerella forsythia*, were both detected in the supra-gingival plaque of 86.2% of patients and in the gingival crevicular fluid of 80 and 86.7% of patients, respectively. Certain microorganisms were statistically more frequently detected in patients with digestive disorders and taking certain medications.

**Discussion:**

The oral microbiota composition of the adults with ASD showed significant differences compared to neurotypical individuals, particularly in the prevalence of the specific microorganisms *P. gingivalis*, *T. tenax* and *E. gingivalis* ST1. The detection frequency of periodontitis and periodontopathogens may have been underestimated due to the lack of cooperation of the adults with ASD during clinical examination and microbiota sampling. Further studies on larger cohorts are needed to consolidate these results to gain a better understanding of variations in oral microbiota.

## 1 Introduction

Autism Spectrum Disorder (ASD) is a neurodevelopmental disorder affecting 700,000 people in France. A third of these subjects have an associated intellectual disability and some have other co-morbidities, such as epilepsy, hyperactivity, anxiety, depression, sleeping, learning or attention disorders, as well as gastrointestinal disorders (Restrepo et al., 2020). To date, the etiology of ASD remains unknown and no consensual treatment has been shown to be effective. In addition to a genetic predisposition associated with exposure to one or more environmental factors, the recent involvement of abnormalities in the intestinal microbiota in certain subjects with ASD opens up new etiopathogenic and therapeutic perspectives that remain to be explored. Buffington et al. showed that transferring fecal microbiota modifies the social behavior of mice: the transfer of fecal microbiota from “control mice” to germ-free mice with a social interaction deficit normalized the latter’s behavior (Buffington et al., 2016). Moreover, Sharon et al. demonstrated that transferring intestinal microbiota from a subject with ASD to a germ-free mouse led to a reduction in social interaction and repetitive behavior, which is observed in subjects with ASD (Sharon et al., 2019). Lastly, Kang et al. showed that fecal transfer from healthy donors to subjects with ASD led to an improvement in gastrointestinal symptoms and autistic behavior (Kang et al., 2017, 2019).

The relationship between oral bacteria causing periodontal disease and certain systemic diseases has been demonstrated, either through the direct action of certain bacteria or through an inflammatory response induced by them. A bidirectional link between periodontal disease and diabetes has been shown via the production of pro-inflammatory cytokines (Bui et al., 2019; Genco and Sanz, 2020). Similarly, a relationship has been observed between the presence of certain periodontopathogens and the severity of certain mental disorders, such as Alzheimer’s disease via *Porphyromonas gingivalis,* the keystone pathogen of periodontitis (Maitre et al., 2020). Subjects with ASD often have intestinal and immune system disorders that can lead to chronic inflammation in which periodontal bacteria could potentially play a role, similar to the established link between gut microbiota dysbiosis and ASD, and ultimately influence their behavior (Srikantha and Mohajeri, 2019). Studies have found a different oral microbiota in autistic children compared to neurotypical children (Hicks et al., 2018; Evenepoel et al., 2024; Manghi et al., 2024; Tao et al., 2024), which could be involved in autistic behaviors, as demonstrated in an animal model (Qiao et al., 2022). Qiao et al. observed *Porphyromonas* and *Tannerella* species -two major pathogens in periodontal diseases- in a different proportion in mice which received oral microbiota from ASD donors compared to mice which received oral microbiota from neurotypical donors (Qiao et al., 2022).

People with ASD may have sensory impairments, such as tactile hypersensitivity (American Psychiatric Association, 2013). Dental treatment is an invasive procedure for people who are intolerant of physical contact, which is why it is often difficult to achieve good cooperation during dental care. People with autism will open their mouths, but for a limited period of time and they request regular breaks; sometimes the accompanying persons have to restrain them in the dental chair so that the treatment can be carried out. All these difficulties limit the possibility of assessing the periodontal condition of these patients and explain why, in the literature, periodontal disease in subjects with ASD is mostly assessed using a visual periodontal parameter indicating the level of gingival inflammation only, and mainly concerns children and adolescents (Fakroon et al., 2015; Da Silva et al., 2017). Periodontal diseases are a worldwide burden. The prevalence of severe periodontal disease is 19% in people over 15 years old (World Health Organization, 2022). Fakroon et al. concluded that children with ASD showed significantly higher periodontal treatment needs than neurotypical children (Fakroon et al., 2015). In the absence of appropriate treatment, it is likely that the periodontal condition of these children in adulthood will have deteriorated. To our knowledge, no study has evaluated the periodontal status, particularly the presence of periodontal pockets and the associated microbiota, in adults with ASD. Most studies only included children and young adults and did not carry out an accurate clinical examination to detect periodontal pockets, which are infectious sites and leading to tissue loss surrounding teeth.

In this pilot clinical study, we aimed to assess the periodontal and dental status of adults with ASD with accurate parameters, and the presence of microorganisms associated with periodontal diseases.

The first hypothesis tested in adults with ASD was the high level of supra-gingival plaque and consequently, the presence of periodontal disease. The second hypothesis was that adults with ASD have a different microbial composition of certain microorganisms than neurotypical patients, with mostly periodontopathogens.

## 2 Materials and methods

### 2.1 Study design

This clinical study was a cross-sectional, epidemiological, observational study describing the periodontal and dental status of a population of adults with ASD and their associated oral microbiota. The study was conducted over 5 months (11/2023 to 04/2024) on patients treated in the dentistry department (specialized care unit) of the University Hospital of Nancy and living in residential nursing homes.

The study was approved by the Ethical Committee of the University Hospital of Nancy (No. ID-RCB: 2023-A01024-41). The study protocol was submitted to the ClinicalTrials.gov website and was assigned the reference NCT05910424.

### 2.2 Study population

Of the 34 adults with ASD, only 30 were included in the clinical study.

Inclusion criteria were:

- Consent from the legal guardian obtained.
- Autistic adults, male or female, aged between 18 and 60.

Non-inclusion criteria were:

- Aged under 18.
- Antibiotics or probiotics taken within the 3 months prior to the study.
- Smoking habits.
- Periodontal treatment received in the 6 months prior to sampling.

Non-inclusion factors or lack of cooperation was the cause for rejecting four subjects.

All subjects included in this study were diagnosed with ASD according to the DSM-5 (5th edition of the Diagnostic and Statistical Manual of Mental Disorders) by a psychiatrist. ASD is a neurodevelopmental disorder based on 5 criteria of which the two main ones are a persistent deficit in communication and social interaction in multiple contexts, and reduced repetitive behaviors and limited interests and activities. Subjects with ASD may also have sensory impairments, such as tactile hypersensitivity (American Psychiatric Association, 2013).

### 2.3 Data collection

#### 2.3.1 Demographic data, medical history and lifestyle habits

With the agreement of the legal guardians, the demographic and medical data were collected via a questionnaire (medical history, drug treatments, allergies, digestive and sleep disorders, snacking, oral hygiene habits, dental consultations and patient cooperation).

#### 2.3.2 Clinical protocol -periodontal and dental parameters

The Dutch Periodontal Screening Index (DPSI) was used to assess the periodontal status, i.e., measure the depth of the gingival sulcus or periodontal pocket using a periodontal probe, evaluate the presence of gingival recession, bleeding, calculus or other retention factors, and determine 4 status (Van der Velden, 2009): healthy periodontium, gingivitis (bleeding on probing), periodontitis with probing depth less than 6 mm and periodontitis with probing depth greater than or equal to 6 mm (corresponding to stages 3 and 4 of the 2017 Chicago classification, a global consensus defining periodontal disease) (Papapanou et al., 2018). However, only buccal probing was carried out in this study due to the limited cooperation of some patients. Oral hygiene was defined according to the plaque index of Silness and Loe (1964). In our study, the highest plaque index per sextant was recorded.

Dental condition was characterized according to the DMFT index (Decayed-Missing-Filled-Tooth). The DMFT score, used as a reference for oral check-ups, provides the number of decayed, missing, and filled teeth of each individual, as well as the percentage of the patient population with at least one decayed tooth, at least one missing tooth and at least one tooth treated, excluding wisdom teeth (Haute Autorité de Santé, 2010).

To assess the periodontal flora, two samples were taken:

- The supra-gingival dental plaque was collected using a William probe at two dental sites, one in the maxilla and one in the mandible, where the amount of plaque was most abundant. The samples were pooled in empty, encoded, single, sterile Eppendorf tubes.
- The gingival crevicular fluid was collected to obtain a subgingival biofilm. To do so, four sterile paper strips, two in the maxilla and two in the mandible where the gingiva was seen to be the most inflamed, were inserted for at least 5 s. All the paper points were then pooled in empty, encoded, single, sterile Eppendorf tubes.

All samples were stored at −80°C until analysis.

#### 2.3.3 Microbial identification and quantification

Microbial DNA was extracted and purified from the paper points with the QIAamp DNA mini kit® (Qiagen, France), according to the manufacturer’s instructions. The quantity and purity of microbial DNA obtained were assessed spectrophotometrically using the Nanodrop™ 2000C (ThermoScientific, Waltham, MA, USA). The bacteria and protozoa most frequently detected in periodontal disease were sought.

The 11 most periodontopathogenic bacteria [bacteria belonging to the Socransky’s red complex (*P. gingivalis*, *T. denticola,* and *T. forsythia*), some belonging to the orange and green complexes (*Prevotella intermedia, Parvimonas micra, Eubacterium nodatum, Fusobacterium nucleatum, Campylobacter rectus, Eikenella corrodens* and *Capnocytophaga* species, respectively) and *Aggregatibacter actinomycetemcomitans*] were detected using a semi-quantitative PCR, MicroIDent Plus 11 kit (Hain LifeScience, Nehren, Germany). All bacterial species were detected with a minimum threshold of 10^4^ bacteria except for *A. actinomycetemcomitans* with a minimum threshold of 10^3^ bacteria, as specified by the manufacturer. The primers used to detect the bacteria are the property of Hain Lifescience and are therefore an industrial secret. However, the accuracy of these primers has been assessed by Santigli& al. These authors have shown the high specificity of MicroIDent kit (Santigli et al., 2016).

Briefly, 2.5 μL of extracted DNA was mixed with 22.5 μL of a solution containing specific primers, nucleotides, buffer and Taq Polymerase, as previously described (Urbán et al., 2010). PCR cycles first included a denaturation step at 95°C for 5 min. Followed by 10 cycles at 95°C for 30 s. and at 58°C for 2 min., 20 cycles at 95°C for 25 s., at 52°C for 40 s. and 70°C for 40 s., and a final extension step at 70°C for 8 min. The amplicons were then hybridized according to the manufacturer’s recommendations: biotin-labeled amplicons were denatured and incubated at 45°C for 30 min. With hybridization buffer containing a complementary probe specific for each targeted bacterial species. A washing step removed non-specifically bond DNA. Streptavidin conjugated alkaline phosphatase was added, the strips were rinsed and hybridation products were revealed by adding dimethyl-sulfoxide. The interpretation of the colorimetric band intensity was carried out by two independent individuals.

The QuantiNova® Multiplex kit (Qiagen, France) was used according to the manufacturer’s instructions to identify protozoa. TaqMan PCR amplification was performed for *Entamoeba gingivalis* ST1 and ST2 and *Trichomonas tenax*, as previously described and validated (Dubar et al., 2019; Zaffino et al., 2019). Briefly, the primers were designed to amplify a 153pb fragment inside the SSU rDNA of *E. gingivalis.* Two TaqMan probes were selected specifically for *E. gingivalis* subtype 1 or 2 (variant kamaktli) (ST1 and ST2), respectively. Each 20 μL reaction consisted of 17.5 μL of the solution and 2.5 μL of the extracted DNA. All primers and TaqMan are detailed in Table 1.

**Table 1:** Specificity of the primers and probes for protozoa used in this study.

Primer set / probe		Sequence	Product size (pb) /target	Fluorophore
Primers	EG1F	5′-TACCATACAAGGAATAGCTTTG-3′	153 pb of SSU rDNA of *E. gingivalis*	
	EG2R	5′-GATATTTTCATTGATTCCTTGTC-3′		
TaqMan probe	EG12P	5′-FAM-GAATAGGCGCATTTCGAACAGGA-BHQ1	*E. gingivalis* ST1	FAM
	EG12PK	5′-HEX-AGTTGTTTGTACAAGTGGGCGCAT-BHQ1	*E. gingivalis* ST2	HEX
Primers	TT5PS	5′-GGAGTTGCATACATCATGAC-3′	163 of 5.8 S rDNA of *T. tenax*	
	TT5PAS	5′-CGTATAGCAGACAACGTAAGT-3′		
TaqMan probe	TT5S	5′-HEX-CTAAACTTGGCTTCGGCTGAGAA-BHQ2	*T. tenax*	ROX

For all PCR reactions, the thermocycler BioRad CFX96 was used under the following conditions: 95°C for 15 min. of TaqMan polymerase activation, 45 cycles for the denaturation step at 94°C for 15 s., followed by a combined hybridization and elongation at 60°C for 60 s.

### 2.4 Statistical analysis

According to data collected from the general population, the main periodontopathogen, *P. gingivalis*, was found in 25% of the subjects without periodontal disease and in 79% of subjects with periodontitis (Griffen et al., 1998). We hypothesized that if *P. gingivalis* was found in 25% of patients, in a population of 40 subjects, at least 5 subjects with a 95% confidence interval would test positive for the bacterium. However, only 30 patients were included in this study due to the lack of cooperation of some subjects.

For the descriptive analysis, the qualitative variables were reported by frequency of occurrence, expressed as a percentage, and the quantitative variables by median and IQR (Q1 and Q3) as the distribution did not follow the normal distribution.

For the statistical analysis, the Fischer exact test was used for qualitative variables and the non-parametric Wilcoxon test for quantitative variables.

Statistics were calculated using SAS 9.4 software (SAS Institute, Inc., Cary, NC, USA). The significance threshold was set at *p* < 0.05 for all tests.

## 3 Results and discussion

### 3.1 Population characteristics

The study included 30 adults with ASD: 21 males (70%) and 9 females (30%) with a median age of 31 years. These data are consistent with the literature describing the gender distribution in an autistic population as 2/3 male and 1/3 female (Loomes et al., 2017).

The medical history of the adults with ASD is summarized in Table 2. The population suffered from severe autism spectrum disorders with 75% of the subjects taking neuroleptic drugs and 32% treated for epilepsy. Thirty-nine percent (n = 11/28) of individuals suffered from digestive problems, which was less than reported in the literature, and had at least one of the following symptoms: diarrhea, constipation (maximum of three stools per week) or abdominal pain. In this study, diarrhea and constipation were less (7%, n = 2/28 vs. 13%) and more frequent (32%, n = 9/28 vs. 22%), respectively, than the results found by Holingue et al. (2018). They concluded that the gastrointestinal symptoms differed significantly depending on the age of individuals, the study design and the study sample. Other factors should also be considered: neuroleptic drugs, for example, known to affect the intestinal transit due to their anticholinergic effect, could be one of the main causes of constipation. Moreover, 14% (n = 4/28) of adults with ASD suffered from abdominal pain, which was in agreement with the results of Holingue et al. (2018): this result may have been underestimated because of pain circuitries and perception impairment in adults with ASD. Sleeping disorders, another clinical sign characterizing autism, affected 41% of the subjects.

**Table 2:** Medical history of autist patients (N = 28).

Co-morbidities		N	%
Diarrheas	Missing	2	
	No	26	92.9
	Yes	2	7.1
Constipation	Missing	2	
	No	19	67.9
	Yes	9	32.1
Abdominal pain	Missing	2	
	No	24	85.7
	Yes	4	14.3
Sleeping trouble	Missing	3	
	No	16	59.3
	Yes	11	40.7
Epilepsy	No	22	73.3
	Yes	8	26.7
Anti-epileptics	Missing	2	
	No	19	67.9
	Yes	9	32.1
Neuroleptics	Missing	2	
	No	7	25.0
	Yes	21	75.0
Benzodiazepines and benzodiazepine-like agents	Missing	2	
	No	22	78.6
	Yes	6	21.4
Anti-parkinson drugs	Missing	2	
	No	21	75.0
	Yes	7	25.0
Antidepressants	Missing	2	
	No	24	85.7
	Yes	4	14.3

Regarding lifestyle habits, all adults with ASD had regular check-ups with a dental surgeon, with varying degrees of cooperation: only 57% (n = 16/28) of them showed rather good cooperation, enabling care to be carried out. For the others, appropriate procedures were necessary, such as restraint, drug sedation or conscious sedation, or care under general anesthesia. Carers had to maintain them in the dental chair and ensure they did not move during treatment. All brushed their teeth daily, 2 or 3 times a day, mainly with a manual toothbrush (72.4%, n = 21), either on their own (41.4%, n = 14) or with the help of someone else (58.6%, n = 12), and generally cooperated well during this procedure.

### 3.2 Dental and periodontal status

The average DMFT index, characterizing oral health, was 4.0 (Q1: 1.0; Q3: 5.0), and only one subject needed cavity treatment. A bivariate analysis showed, as expected, a significant increase in the DMFT index with age (Spearman correlation = 0.53, *p* = 0.002). Our results contradicted those in the literature, which describes a higher DMFT index in autistic populations (Corridore et al., 2020; Pi et al., 2020). This difference could be explained by patients attending regular dental check-ups at least once a year, controlled snacking and daily supervision by care staff. All of the adults with ASD in our cohort, except one, were full-time residents in a specialized care facility. As observed by Shapira et al. (1989), institutionalized adults with ASD had lower caries rate than those living at home. Lastly, the adults with ASD in this study had a lower DMFT index than French neurotypical adults, which varies between 13 and 15, with at least one untreated cavity in 33 to 50% of them (Haute Autorité de Santé, 2010).

Regarding periodontal status, only 13.3% of subjects had a healthy periodontium, 60% had gingivitis and 26.7% had periodontitis (Table 3). It is challenging to directly compare our results with those of Blomqvist et al. (2015) regarding the frequency of gingivitis detection due to methodological differences; the authors used a percentage of bleeding on probing of all the teeth of adults with ASD. However, it would appear that their population had a low percentage of gingival bleeding and therefore little gingivitis. A systematic review and meta-analysis by Da Silva et al. (2017) showed that autistic children and young adults had an average of 69% gingivitis, a figure similar to that found in our study. The prevalence of adults with ASD with periodontal pockets ≥6 mm is similar to that of the French adult neurotypical population in the 35–39 age group, as reported by Bourgeois et al. (2007), but lower than found by Carra et al. (2018) in population with an average age of 46 years (6.5% vs. 4.3 and 34%, respectively).

**Table 3:** Periodontal parameters of autist adult patients.

Periodontal parameters	Scale	N	%
Periodontal status according to DPSI index	Healthy	4	13.3
	Gingivitis	18	60.0
	Periodontal pockets <6 mm	6	20.0
	Periodontal pockets ≥6 mm	2	6.7
Plaque index (Silness & Löe)	0 = Absence of dental plaque	1	3.3
	1 = Plaque visible with a probe	9	30.0
	2 = Plaque visible	13	43.3
	3 = Abundant Plaque	7	23.3

The low prevalence of periodontitis was surprising given the large amount of plaque on the teeth of almost all the patients included. The assessment of the quantity of supra-gingival plaque showed that only one patient (3.3%) exhibited adequate teeth brushing as no plaque was found, nine subjects (30%) had plaque visible on the probe and the majority of patients (66.6%) had a quantity of visible plaque or even abundant on at least one tooth, showing that oral hygiene could be improved. These results are consistent with the Da Silva et al. (2017) meta-analysis, which showed that the majority of autistic children or young adults have dental plaque that is visible or in abundant quantities.

Moreover, the quantity of dental plaque detected in adults with ASD in this study was largely underestimated because only the buccal surfaces of the teeth were examined. As observed in neurotypical patients, the lingual surfaces of the mandibular teeth may be poorly cleaned (Söder et al., 2003).

### 3.3 Oral microorganisms

Microorganisms in supra-gingival and crevicular samples were collected and some were identified. Both periodontal bacteria and oral protozoa were detected in the different samples. One patient was not sampled because he presented no inflammatory sites and no supra-gingival plaque.

Regarding periodontal bacteria, the detection frequencies of all the periodontal bacteria of interest are summarized in Table 4: two major periodontal pathogens belonging to the red complex were frequently detected: *T. denticola* and *T. forsythia* were identified in 86.2% of plaque samples, and in 80 and 86.7% of crevicular samples, respectively. Bacteria of the green complex (*E. corrodens*, *Capnocytophaga* spp.) and one belonging to the orange complex (*F. nucleatum*) were detected in the supra-gingival plaque of all patients and in the plaque of 96.2% of patients for *P. micra*.

**Table 4:** Percentage and quantities of periodontal bacteria from supra gingival plaque and crevicular fluid samples of patients with ASD.

	Frequency of the quantity of periodontal bacteria detected									
Species	Supra-gingival plaque (N = 29) (%)					Gingival crevicular fluid (N = 29) (%)				
	−	(+)	+	++	+++	−	(+)	+	++	+++
*A.actinomycetem -comitans*	79.3	6.9	0.0	3.4	10.3	83.3	10.0	0.0	0.0	6.7
*P. gingivalis*	62.1	6.9	10.3	0.0	20.7	66.7	6.7	3.3	6.7	16.7
*P. intermedia*	62.1	10.3	10.3	6.9	10.3	60.0	13.3	13.3	3.3	10.0
*T. forsythia*	13.8	3.4	17.2	24.1	41.4	13.3	16.7	3.3	13.3	53.3
*T. denticola*	13.8	3.4	27.6	44.8	10.3	20.0	16.7	6.7	30.0	26.7
*P. micra*	13.8	31.0	44.8	3.4	6.9	33.3	33.3	30.0	3.3	0.0
*F. nucleatum*	0.0	0.0	3.4	31.0	65.5	10.0	0.0	3.3	46.7	40.0
*C. rectus*	37.9	13.8	24.1	13.8	10.3	23.3	10.0	26.7	23.3	16.7
*E. nodatum*	75.9	3.4	17.2	3.4	0.0	63.3	23.3	13.3	0.0	0.0
*E. corrodens*	0.0	6.9	27.6	41.4	24.1	16.7	6.7	33.3	40.0	3.3
*Capnocytophaga spp*	0.0	0.0	6.9	10.3	82.8	3.3	6.7	6.7	30.0	53.3

The hybridation on membrane strips give different signal strengths which have been assigned for all bacteria, according to the following code: (−) <10^4^ (except *A.a*: <10^3^), (+) 10^4^ (except *A.a*: 10^3^), + <10^5^ (except *A.a*: <10^4^), ++ <10^6^ (except *A.a*: <10^5^), +++ ≥10^6^ (except *A.a*: ≥10^5^).

In the gingival crevicular fluid, the bacteria most frequently detected were the same as those found in the supra-gingival plaque, i.e., *Capnocytophaga* spp. in 96.7%, *F. nucleatum* in 90%, *T. forsythia* in 86.7%, *E. corrodens* in 83.3%, *T. denticola* in 80%, *C. rectus* in 76.7% and *P. micra* in 66.7% of the crevicular fluid samples of the patients.

The bacterial identification results were difficult to compare with those of other studies as the samples taken were from different sites (tongue and/or saliva samples, etc.), the population consisted of children and adolescents, and the microbiota was explored using high-throughput sequencing, at the phylum and family level, and not always at the species level (Evenepoel et al., 2024; Qiao et al., 2018; Hicks et al., 2018). Nevertheless, the *Tannerella* genera were significantly more abundant in children with ASD than in neurotypical children (Evenepoel et al., 2024). In this study, the detection frequency of *T. forsythia* was similar to that of neurotypical adults, evaluated previously by our team using similar technical procedures (Dubar et al., 2020). In our study, *P. gingivalis* and *F. nucleatum* were detected less frequently in the periodontal pockets of adults with ASD than in neurotypical adults compared to Dubar’s study using the same analysis (33.3% [17.3–52.8%] vs. 70, and 90% [73.7–97.9%] vs. 98.3%, respectively) (data not shown) (Dubar et al., 2020). These results were consistent with those of Qioa et al., who showed that the genera *Porphyromonas* and *Fusobacterium* were reduced in children with ASD (Qiao et al., 2018). However, these results should be interpreted with caution because the collection time of our protocol was shorter (5–10 s versus 30 s), which limited the amount of DNA collected, and the population recruited had less severe periodontitis than those in Dubar’s study. Furthermore, the periodontal bacteria detection frequency in the oral cavity of adults with ASD was underestimated as only the pathological sites on the buccal surfaces of the teeth were sampled.

**Figure 1:**
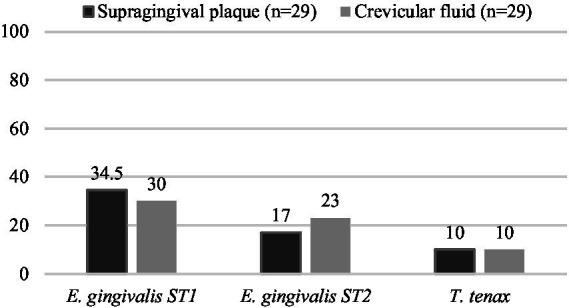
Detection frequencies of *E. gingivalis ST1* & *ST2* and *T. tenax* in supra-gingival plaque and crevicular fluid samples in autistic patients.

Protozoa were less frequently detected than periodontal bacteria in both supra-gingival plaque and crevicular fluid. The most frequently identified protozoan was *E. gingivalis* ST1 in 34.5% of supra-gingival plaque and 30% of crevicular patient samples. The detection frequencies of oral protozoa are summarized in Figure 1. The percentage of protozoan detection in crevicular fluid was lower for *E. gingivalis* ST1 and for *T. tenax* than in the study by Dubar et al. (30% [14.7–49.4%] vs. 70, and 10% [2.1–26.5%] vs. 33.3%, respectively). This difference may be due to the short collection time, but also to the fact that the subjects of the study by Dubar et al. (2020) had more severe periodontal disease and therefore an anaerobic environment that was probably more conducive to the development of protozoa: the deeper the periodontal pocket, the lower the level of O_2_ and pH in the subgingival biofilm, and the higher the level of metabolic products, which facilitates the development of anaerobic microorganisms (Abdulkareem et al., 2023; Borisy and Valm, 2021).

No highly strong association was observed between protozoa and bacteria, or between different bacterial species (Table 5). A strong association was observed between *F. nucleatum* and *T. forsythia*, and to a lesser extent, between *T. denticola* and *T. forsythia*, and *F. nucleatum* and *E. corrodens* in crevicular fluid samples (Phi coefficient 0.84, 0.78 and 0.74, respectively, *p* < 0.05). A moderate association between *P. gingivalis* and *T. tenax* was detected, which was consistent with our previous findings in neurotypical patients (Dubar et al., 2019). However, although the co-detection of *E. gingivalis* ST1 and *P. micra*, *E. nodatum* and *C. rectus* observed in this study was not found in Dubar’s results (Phi coefficient 0.46, 0.41 and 0.36, respectively, *p* < 0.05), these associations were due to metabolic and/or adhesion cooperation in the biofilm, as previously described (Sedghi et al., 2021).

**Table 5:** Comparison of the association between different bacteria and between bacteria and protozoa from crevicular fluid and supra-gingival plaque samples.

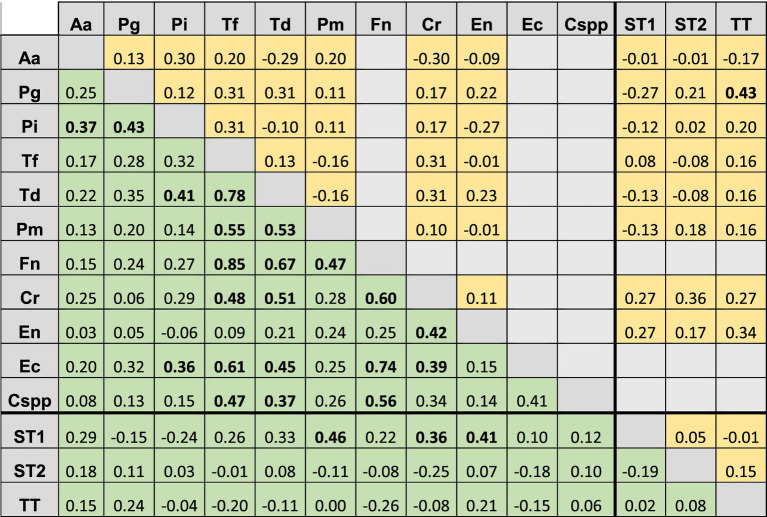

Phi coefficient: a strong association between 2 variables is considered to exist when the coefficient is >0.0 or < −0.80. Significant associations are shown in bold (*p* < 0.05).

■crevicular fluid, ■ supra-gingival plaque.

### 3.4 Factors associated with microorganism detection

The detection frequency of microorganisms according to periodontal status is described in Table 6. *T. tenax* was only detected in dental plaque samples from patients with periodontitis. The amoeba *E. gingivalis* ST2 was mainly detected in periodontal pockets <6 mm, as previously described by Dubar et al. (2019). *E. gingivalis* ST2 was identified in crevicular samples of both gingivitis and periodontitis patients, but not in healthy patients. The periodontopathogen *A. a*, was mainly detected in samples from periodontitis patients, but never in healthy patients. According to the plaque index, *T. tenax* in both dental plaque and crevicular fluid samples was significantly detected only when the plaque was very abundant (*p* = 0.015 and *p* = 0.014, respectively), which was consistent with a previous study showing the high prevalence of the flagellate in patients with bad oral hygiene (Bisson et al., 2018). *P. gingivalis* in both dental plaque and crevicular fluid samples was mainly detected in the presence of very abundant plaque (data not shown). Surprisingly, for all other bacteria, no significant differences were observed between plaque or crevicular fluid samples from healthy patients, or patients with gingivitis or periodontitis. As explained above, these results could be due to a too-short sampling time.

**Table 6:** Periodontal status according to presence of bacteria and protozoa in supra-gingival plaque and crevicular fluid samples.

	Total N = 30		Healthy N = 4		Gingivitis N = 18		Periodontitis. PPD < 6 N = 6		Periodontitis. PPD ≥ 6 N = 2		
	N	%	N	%	N	%	N	%	N	%	*p*
Detection of *Trichomonas tenax* in supra-gingival plaque samples											0.025
No	26	89.7	4	100	17	100	4	66.7	1	50.0	
Yes	3	10.3	0	0	0	0	2	33.3	1	50.0	
Missing	1				1						
Detection of *A. actinomycetemcomitans* in crevicular fluid samples											0.031
No	25	83.3	4	100	17	94.4	3	50.0	1	50.0	
Yes	5	16.7	0	0	1	5.6	3	50.0	1	50.0	
Detection of *Entamoeba gingivalis* ST1 in crevicular fluid samples											0.033
No	21	70.0	4	100	14	77.8	3	50.0	0	0	
Yes	9	30.0	0	0	4	22.2	3	50.0	2	100	
Detection of *Entamoeba gingivalis* ST2 in crevicular fluid samples											0.068
No	23	90.0	4	100	17	94.4	2	33.3	2	100	
Yes	3	10.0	0	0	1	5.6	4	66.7	0	0	

Test exact of Fisher. *p* < 0.05. (PPD, periodontal pocket depth).

Certain bacteria and protozoa were significantly associated with other medical pathologies (Tables 7, 8). Specific bacteria in crevicular fluid, such as *P. intermedia*, were less frequently detected in adults with ASD with constipation (*p* = 0.01) while others, such as *E. nodatum*, were more frequently detected in adults with ASD on anti-epileptic drugs (*p* = 0.01). Regarding the prevalence of protozoa, *E. gingivalis* ST1 in both crevicular fluid and supra-gingival plaque was more frequently detected in adults with ASD with constipation and digestive disorders, and on anti-epileptic drugs (*p* = 0.013, *p* = 0.01, *p* = 0.05, *p* = 0.04, *p* = 0.009 and *p* = 0.001, respectively). Benzodiazepine treatment also impacted the presence of certain microorganisms, such as *E. nodatum* and *E. gingivalis* ST1 (*p* = 0.022, *p* = 0.038). These results may be due to the side effects of certain drugs; benzodiazepines and anticonvulsant drugs are known to reduce saliva flow (Fortuna et al., 2023). Moreover, anticonvulsant drugs are also known to induce gingival enlargement. These side effects can modify the environment and therefore interfere with the colonization and development of certain micro-organisms. Patients with hyposalivation are more susceptible to mucosal infection due to both buccal flora changes and salivary flow reduction, which weakens the mechanical protection of the physiological cleaning of dental surfaces and oral mucosa, the innate immune responses and the buffering capacity (Ligtenberg and Almståhl, 2015). Studies have shown that xerostomia/ hyposialia modifies the composition of buccal bacteria (Wade, 2021; Hayashi et al., 2015). Lastly, the specific variables of changes in the oral microbiota cannot be highlighted which raises the question: are they a specific microbial signature of autism or simply a consequence of multiple factors, medication being a relevant one in severe forms of autism disorders?

**Table 7:** Association between *E. gingivalis ST1* in supra-gingival plaque samples and certain systemic disease in autistic patients.

*E. gingivalis* ST1	Absence n = 19 (65.5%)		Presence n = 10 (34.5%)		
	N	%	N	%	*p*
Constipation					
No	14	82.4	4	40	0.039
Yes	3	17.6	6	60	
Missing	2		-		
Digestive disorders					
No	13	76.5	3	30	0.040
Yes	4	23.5	7	70	
Missing	2		-		
Epilepsy					
No	17	89.5	4	40.0	0.009
Yes	2	10.5	6	60.0	
Missing	2		-		
Anti-epileptic drug					
No	16	88.9	2	22.2	0.001
Yes	2	11.1	7	77.8	
Missing	1		1		

Test exact of Fisher. *p* < 0.05.

**Table 8:** Association between bacteria and protozoa in crevicular fluid plaque samples and certain systemic disease in autistic patients.

*P. intermedia*	Absence (n = 18; 60.0%)		Presence (n = 12; 40.0%)		
	N	%	N	%	*p*
Constipation					
No	9	50.0	10	100	0.010
Yes	9	50.0	0	0	
Missing	-		2		
*E. nodatum*	Absence (n = 19; 63.3%)		Presence (n = 11; 36.7%)		
Epilepsy					
No	17	89.5	5	45.5	0.027
Yes	2	10.5	6	54.5	
Anti-epileptic					
No	15	88.2	4	36.4	0.010
Yes	2	11.8	7	63.6	
Missing	2		-		
Benzodiazépines and related drugs					
No	16	94.1	6	54.5	0.022
Yes	1	5.9	5	45.5	
*E. gingivalis* ST1	Absence (n = 21; 70%)		Presence (n = 9; 30%)		
Constipation					
No	16	84.2	3	33.3	0.0127
Yes	3	15.8	6	66.7	
Digestive disorders					
No	15	78.9	2	22.2	0.0104
Yes	4	21.1	7	77.8	
Epilepsy					
No	18	85.7	4	44.4	0.0318
Yes	3	14.3	5	55.6	
Anti-epileptic drug					
No	17	85	2	25	0.0048
Yes	3	15	6	75	
Benzodiazepines					
No	18	90	4	50	0.0384
Yes	2	10	4	50	

Test exact of Fisher. *p* < 0.05.

### 3.5 Study limitations

This pilot study assessed the periodontal condition of adults with ASD using the DPSI index, which is a good compromise for diagnosing the presence of periodontal disease in a short period of time in order to preserve patient cooperation. However, it should be noted that it is less accurate than full periodontal charting. Consideration could be given to supplementing the clinical examination with X-rays to assess bone loss more accurately. The prevalence of periodontal disease in our population was underestimated due to the absence of a clinical examination of the palatal and lingual surfaces of the teeth (assessment of gingival inflammation, presence of periodontal pockets, quantity of supra-gingival dental plaque, etc.). The same observation can be made about microbial detection: the short sampling time and the impossibility of sampling the palatal and lingual areas of the teeth are factors that did not allow us to draw clear conclusions about the presence of bacteria and protozoa in the oral cavity of adults with ASD. Moreover, it was not always possible to isolate the teeth during sampling; therefore, salivary contamination could not be ruled out, limiting the interpretation of the microorganisms detected.

## 4 Conclusion

The study population was representative of dependent adults for whom severe autistic disorders significantly alter their quality of life. The population had a low DMFT index and a similar percentage of periodontal disease as the neurotypical population of the same age. However, the prevalence of this disease was certainly underestimated due to an incomplete assessment of clinical periodontal parameters. The oral microbiota of adults with ASD appeared to be different for certain microorganisms, such as *P. gingivalis*, *F. nucleatum* and *E. gingivalis* ST1, which were detected less frequently in adults with ASD than in the neurotypical population. Comprehensive longitudinal studies involving larger cohorts are essential to clarify the complex alterations in oral microbiota among adults with ASD, potentially facilitating the development of microbiota-based classification systems analogous to those used for intestinal microbiota, and ultimately paving the way for novel, targeted therapeutic interventions tailored to this unique population.

## Data Availability

The raw data supporting the conclusions of this article will be made available by the authors, without undue reservation.
